# Aesthetical and Functional Rehabilitation for an Ankylosed Maxillary Canine—A Case Report

**DOI:** 10.3390/clinpract14050159

**Published:** 2024-09-27

**Authors:** Tatiana Roman, Maxime Delarue, Matthieu Esquenet, Frédéric Rafflenbeul, Catherine Petit, Naji Kharouf, Olivier Etienne

**Affiliations:** 1 Department of Biomaterials and Bioengeneering, INSERM UMR_S 1121, 67000 Strasbourg, France; 2 Department of Prosthodontics, Faculty of Dental Surgery, Strasbourg University, Hôpitaux Universitaires de Strasbourg, 1 Place de l’Hopital, 67000 Strasbourg, France; 3 Department of Oral Surgery, Faculty of Dental Surgery, Strasbourg University, Hôpitaux Universitaires de Strasbourg, 1 Place de l’Hopital, 67000 Strasbourg, France; 4 Department of Dentofacial Orthopedics, Faculty of Dental Surgery, Strasbourg University, Hôpitaux Universitaires de Strasbourg, 1 Place de l’Hopital, 67000 Strasbourg, France; 5 Department of Periodontology, Faculty of Dental Surgery, Strasbourg University, Hôpitaux Universitaires de Strasbourg, 1 Place de l’Hopital, 67000 Strasbourg, France; 6 Department of Endodontics and Conservative Dentistry, Faculty of Dental Surgery, Strasbourg University, 1 Place de l’Hopital, 67000 Strasbourg, France

**Keywords:** canine ankylosis, case report, cantilever bridge, decoronation, root resorption, occlusal plane cant, ankylosed tooth

## Abstract

**Background:** As the functional and aesthetical importance of the canine cannot be overstated, the management of a missing canine is challenging. This case report describes the treatment of an infra-occluded ankylosed maxillary canine in a patient with previously failed orthodontic treatment. **Case description:** A 20-year-old patient sought a second opinion for orthodontic treatment failure. The patient presented with an impacted, ankylosed, and severely infra-occluded right maxillary canine, as well as an iatrogenic clockwise cant of the maxillary occlusal plane and several root resorptions. The treatment corrected the cant of the occlusal plane while avoiding further root resorption, partially extracted the upper right canine, improved the quality and quantity of the soft tissue in the newly edentulous area, and provided a prosthetic rehabilitation using a lithium disilicate ceramic resin-bonded cantilever bridge. **Conclusions:** The use of a cantilevered bridge resulted in an aesthetically pleasing and minimally invasive rehabilitation. This technique is reversible, does not affect pulp vitality, and is a viable solution for rehabilitating the smiles of young patients. **Clinical significance:** The smile rehabilitation for an ankylosed maxillary canine, especially in the case of a previously failed orthodontic treatment, is an important clinical challenge. A minimally invasive long-term restoration with a cantilever bridge is a viable solution. Functional and aesthetically pleasant results can be achieved with a multidisciplinary approach.

## 1. Introduction

Ankylosis is a pathological phenomenon defined by the fusion of mineralized root surfaces with the surrounding alveolar bone [1,2]. The aetiology of ankylosis encompasses a range of factors, including local metabolic alterations, genetic predisposition, trauma, infections, and previous surgical procedures [1,2]. In clinical practice, ankylosed teeth present with distinctive characteristics, including a metallic sound upon percussion, a lack of mobility, and an infra-occlusion relative to neighbouring teeth [1,2]. Radiographic examination can confirm the diagnostic by demonstrating an interruption of the periodontal ligament space [1,2]. More importantly, the application of orthodontic force fails to induce movement in an ankylosed tooth, emphasising the need for accurate diagnostic approaches.

Accordingly, in the event that an affected tooth fails to exhibit mobility following a three-month course of orthodontic traction, a diagnostic approach should be initiated to ascertain the presence of cervical or root ankylosis [1,2]. Failure to address this promptly may result in occlusal plane canting, particularly when continuous arch traction is employed [1,2].

The maxillary canine is the second most frequently impacted tooth, with an incidence rate of 0.2% to 3% in the general population [1]. Furthermore, canine ankylosis is a common cause of unsuccessful orthodontic treatment, accounting for approximately one-third of cases [1,2]. Given the canine’s pivotal role in smile aesthetics, phonation, and masticatory function, the importance of the maxillary canine is paramount. Nevertheless, the literature lacks detailed descriptions of the management of missing canines, and even less, the management of ankylosed or retained canines [3,4,5]. Accordingly, a therapeutic approach must be selected with careful consideration.

A variety of treatment options are available, including osteotomy in conjunction with orthognathic surgery, auto-transplantation of the canine (in cases of moderate ankylosis), indirect restoration adhesively cemented to the infra-occluded tooth, extraction with orthodontic space closure, extraction with prosthodontic restoration, or even tooth-supported or implant-supported prostheses [3,4,5,6,7,8,9,10,11,12,13,14,15]. However, conventional tooth-supported bridges are an overly invasive procedure, and implant placement may be contraindicated for young patients. It is therefore necessary to consider more conservative approaches.

In 1984, a conservative treatment option for ankylosed teeth was introduced [16]. The procedure is now referred to as decoronation. This technique entails the coronectomy of the ankylosed tooth below the level of the cementoenamel junction, accompanied by the instrumentation of the pulp canal to stimulate bleeding in the periapical region [16]. In post-pubertal patients, the objective of decoronation is to preserve the remaining alveolar bone [6]. The primary indication for this technique is traumatised, ankylosed, and infraoccluded incisors. In the long term, when the patient desires an implant solution, this technique will have preserved the maximum amount of bone volume, thus providing optimal conditions for implant placement. Previous studies have shown that the residual apical fragment is no longer visible 1 to 10 years after surgery [6]. In some cases, root fragments may still be present, but do not interfere with implant placement and healing [17,18]. However, additional guided bone regeneration may be required to increase the horizontal bone ridge [17,18]. By analogy to the incisors, decoronation could be applied to the canine.

The present case report exemplifies the efficacy of a multidisciplinary approach in the case of an ankylosed canine after failed orthodontic treatment. By integrating orthodontic, surgical, and restorative techniques, our team has achieved a harmonious aesthetic and a functional occlusion for the patient. Minimally invasive procedures were instrumental in optimising treatment outcomes.

## 2. Case Description

A 20-year-old female, with no previous medical history, sought a second opinion at the Internal Dento-Facial Orthopaedic Structure, Faculty of Dental Surgery, Strasbourg University, “Hôpitaux Universitaires de Strasbourg”, Strasbourg, France, after failure of orthodontic traction of the right maxillary canine performed by another dentist.

The initial orthodontic treatment had been initiated three years earlier and the patient was displeased with the speed and the aesthetic outcome of the treatment. No data about the situation before the orthodontic treatment were available.

**Figure 1:**
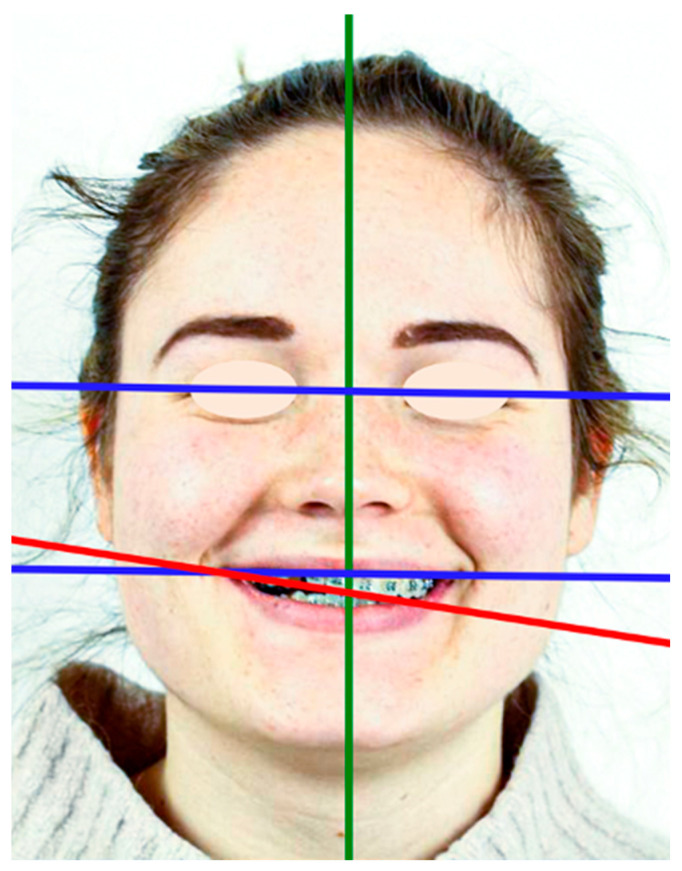
Extra-oral photography of the patient, at the initial appointment with aesthetic analysis. The interpupillary and inter-commissure lines are parallel but not perpendicular to the midsagittal plane; the frontal aesthetic plane is unsightly and presents a clockwise cant. The smile line is low.

**Figure 2:**
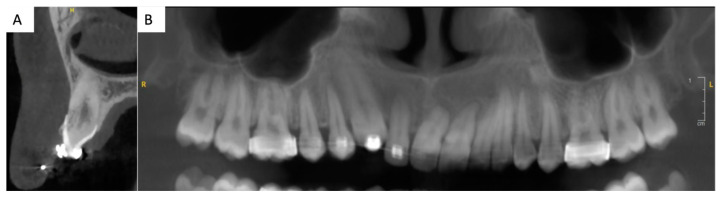
Three-dimensional imaging, CBCT (cone beam computed tomography): (**A**): sagittal section of tooth 13. Thin buccal and palatal cortical bone plates and proximity of the apex of 13 with the right nasal cavity can be noted. (**B**) Two-dimensional reconstruction of the upper jaw. Important root resorptions of the central maxillary incisors can be noted. A root resorption of the lateral incisor is also present.

**Figure 3:**
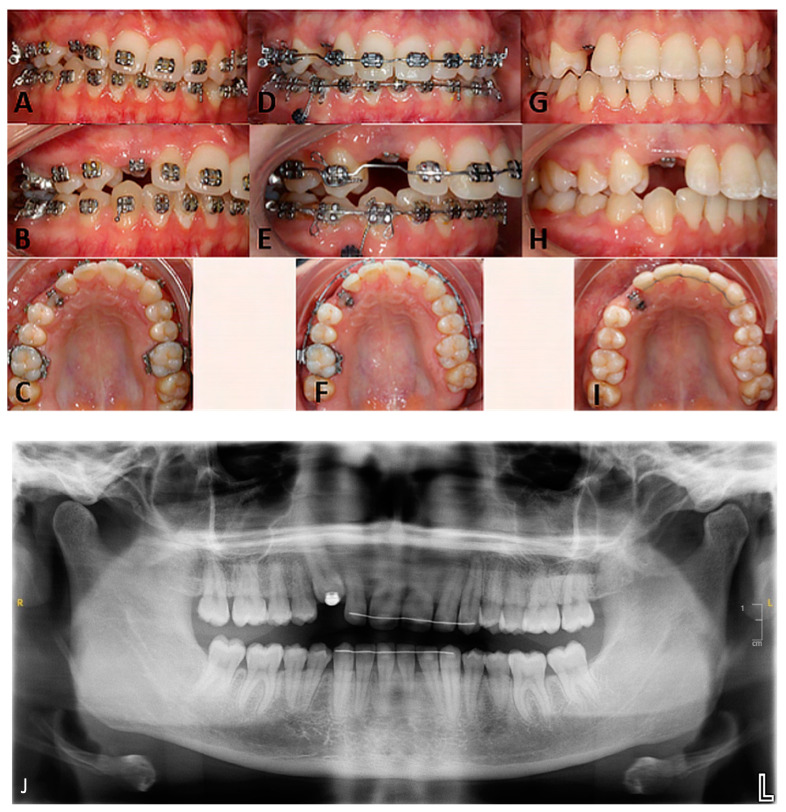
Frontal intraoral views in occlusion, right lateral views, occlusal views of the maxillary arch: (**A**–**C**)—initial situation, at the first consultation; (**D**–**F**)—intermediate situation, during the second orthodontic treatment; (**G**–**I**)—clinical situation upon debonding, (**J**)—post-treatment orthopantomogram.

Upon clinical and radiological examination (Figure 1, Figure 2 and Figure 3), the patient exhibited a harmonious face; a reduced lower facial height; an impacted, ankylosed, and severely infra-occluded right maxillary canine; an upper dental midline deviated 2 mm to the right; a skeletal class II associated with a class II division 1 malocclusion; and an iatrogenic clockwise cant of the maxillary occlusal plane with simultaneous adaptation of the mandibular arch. Several root resorptions were observed, with all maxillary incisors exhibiting signs of this phenomenon. However, the central incisors appeared to have undergone a particularly pronounced degree of root resorption. The clinical diagnosis of ankylosis of # 13 (upper left canine, World Dental Federation notation) was confirmed by the observation of a cant of the occlusal plane, a metallic sound after axial percussion, and a lack of mobility of the canine. A radiographic examination (Figure 2) revealed that the root was centred within the alveolar process. An external resorption with bone replacement was evident in the cervical region on the buccal side of the canine, indicative of tooth ankylosis. In contrast to the thicker palatal cortical bone, the buccal cortical plate was less than one millimetre thick.

In the absence of treatment, an ankylosed, infra-occluded canine with an oblique occlusal plane carries an unfavourable prognosis with regard to both aesthetic and functional outcomes. This condition has the potential to significantly impair the patient’s self-confidence and may also result in the development of temporomandibular joint pain.

A multidisciplinary consultation was convened between the departments of dentofacial orthopaedics, oral surgery, periodontics, and prosthodontics, with the objective of developing an optimal treatment that would honour the patient’s desire for an aesthetic restoration of the smile within a limited timeframe. In consideration of the functional and aesthetic requirements, as well as considering the residual vertical alveolar growth, it was determined that a fixed tooth-supported prosthetic rehabilitation of tooth # 13 was the optimal course of action, given the pre-existing multiple root resorptions [7] (Figure 2). This objective could be achieved through the correction of the occlusal plane cant and partial extraction of the upper right canine, in conjunction with a soft tissue surgery in the newly edentulous area.

**Figure 4:**
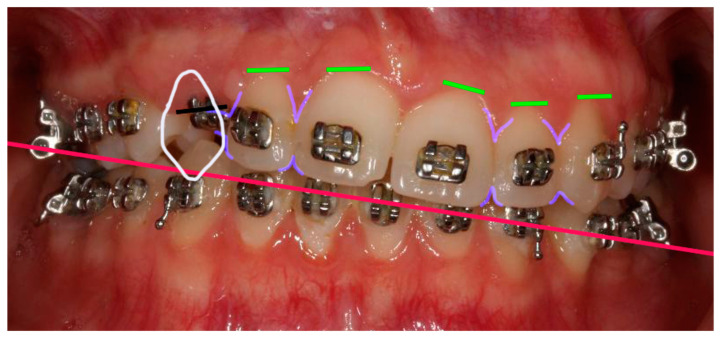
Smile Design. In white, shape of # 23 in position of # 13: the mesio-distal space available for the # 13 tooth is considerably larger than the mesiodistal size of # 23. In lilac, gingival and cervical embrasure spaces: there are considerable discrepancies in the embrasures’ form in the vicinity of # 13. In green, gingival zenith of the anterior teeth showing considerable alignment discrepancies. In red, aesthetic plane, clockwise canted.

A Photoshop Smile Design (PSD, Adobe Photoshop, Adobe, Saint Jose, California, CA, USA) [8] was presented to the patient (Figure 4). The patient was informed of the limitations associated with the present treatment, particularly regarding the aesthetic outcomes. Indeed, the reduced size of the papilla between # 12 and the future # 13, in conjunction with the bone level of the edentulous ridge apical to the cementoenamel of # 12, could have resulted in the persistence of a black triangle. The different mesio-distal diameter of teeth # 13 and # 23 was also considered.

The patient consented to the proposed treatment and the use of all the images for educational and research purposes.

The initial correction of the iatrogenic occlusal plane cant was conducted through orthodontic means, utilising a mandibular temporary anchorage device (TAD) and the ankylosed canine as anchorage points, with the application of extrusion and intrusion mechanics (Aarhus miniscrew, American Orthodontics, Sheboygan, WI, USA; Victory Series brackets, 3M, Saint Paul, MN, USA). The sagittal component of the malocclusion was not addressed in order to reduce the overall treatment time and to avoid exacerbating the existing root resorptions (Figure 3).

After removal of the fixed appliance, an aesthetic temporary solution was implemented using a vacuum-formed retainer (polyethylene terephthalate glycol, Duran, SCHEU-Dental Gmbh, Iserlohn, Germany) in combination with a prefabricated tooth (VITAPAN-Excel, VITA Zahnfabrik, Bad Sackingen, Germany) [8]. Initially, the apical margin of the prosthetic tooth was placed away from the edentulous ridge to avoid any interference with gingival healing during future soft tissue procedures.

**Figure 5:**
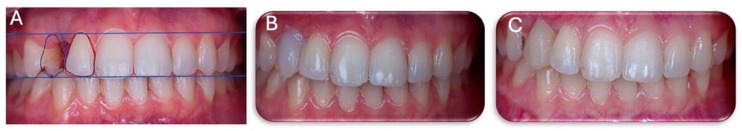
(**A**): Virtual aesthetic project (Smile Design). In black: the current size of tooth # 12 and projection of tooth # 13 with the same dimensions as tooth # 23. In red: Modification of the shape of tooth # 12, optimising the shape of the occlusal and cervical embrasure between tooth # 12 and tooth # 13 and bringing the incisal edge of tooth # 12 back into a harmonious aesthetic plane. In blue: Proposed enlarged shape for tooth # 13 to optimise the shape of the cervical embrasure. (**B**): Mock-up with replacement of tooth # 13 (enlarged) and modification of the shape of tooth # 12. (**C**): Mock-up without modification of the shape of tooth # 12, but with enlarged shape of # 13.

At the same time, a new PSD was performed in the teeth’s final position (Figure 5). Analysis of the smile revealed asymmetry in the cervical and occlusal embrasures between teeth # 12 and # 22, as well as width discrepancies between the mesio-distal width of teeth # 13 and # 23. Indeed, the mesio-distal width of # 13 was larger than the mesio-distal width of # 23. According to the PSD, to enhance the harmony of the patient’s smile, a composite restoration of tooth # 12 could be proposed, focusing on improving the distal transition line, while also enhancing the mesio-distal width discrepancy between the canines. Given the periodontal condition of # 12, this restoration could also help minimise the black triangle between teeth # 12 and # 13.

The following two treatment plans were therefore presented to the patient:

- Rehabilitation of only one tooth, # 13, using a cantilever bridge.
- Rehabilitation of both # 12 and # 13 teeth, using a # 12 veneer and a cantilever bridge.

After diagnostic wax-ups, pre-treatment evaluation was performed (Figure 5). For each treatment plan, the corresponding silicon index was made of vinyl polysiloxane (Harmony, Elsodent, Cergy Pontoise, France) using a wash technique, as previously described [8]. The respective mock-ups were fabricated by using bis-acryl resin (Phoenix MD A2 colour, Elsodent, Cergy Pontoise, France).

Upon carefully considering both options, the patient chose to only proceed with the rehabilitation of the missing tooth.

**Figure 6:**
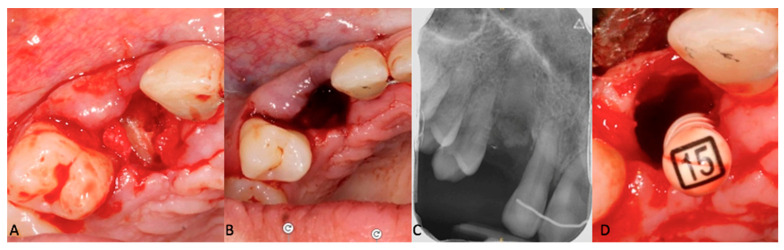
(**A**–**C**): Pre-operative view of the decoronation. (**C**): Post-operative intraoral teleradiograph. (**D**): Pre-operative view of the supra-radicular blood clot formation induced by the remaining pulp parenchyma from the apical fragment.

The next step involved the decoronation of tooth # 13 while aiming to preserve the alveolar ridge. Due to the thin buccal cortical plate (<1 mm) (Figure 2), as well as the position and size of the canine, a full tooth extraction posed a risk of bone damage and could have resulted in an unpleasant gingival defect. To avoid such complications, after removal of the buccal flap, the crown of tooth # 13 was vertically divided into two parts using an orange contra-angle and fissure bur (H162SL.314.014 VPE 1 tungsten bur, Komet, Dublin, Ireland). Both fragments were then extracted, and the remaining root was progressively milled with a handpiece bone ball bur (H141.104.027 VPE 1 tungsten bur, Komet, Dublin, Ireland), while extensive irrigation and optical aids (Orascoptic loupes, Madison, WI, USA) were used to remove all residual enamel. Intra-operative radiographic controls were used to monitor the progress of the decoronation and assess the size of the residual root. An endodontic file (K file, size 15) was used to remove the remaining pulp parenchyma from the apical fragment, inducing intracanal bleeding and promoting supra-radicular blood clot formation (Figure 6). Sutures (5–0, Vicryl Rapide, Raritan, NJ, USA) were applied and the buccal margin was brought closer to the palatal margin to avoid scarring in the aesthetic zone.

**Figure 7:**
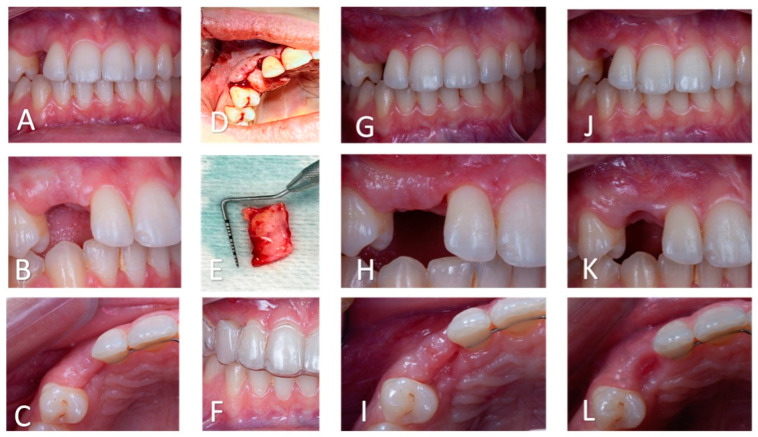
Soft tissue management. (**A**–**C**): Intra-oral views (frontal, lateral, and occlusal, respectively) after decoronation and before periodontal surgery. The soft tissues are thin and the gingival lines of # 13 and # 23 are asymmetric. (**D**): Intra-oral pre-operative view of the mucogingival surgery. Buccal and supra-crestal placement of the graft. (**E**): Connective tissue graft. (**F**): Intra-oral frontal view with the vacuum-formed retainer, worn during healing. (**G**–**I**): Intra-oral views (frontal, lateral, and occlusal, respectively) after periodontal surgery. An increase in the soft tissue thickness in the frontal and transversal planes is observed. (**J**–**L**): Intra-oral views (frontal, lateral, and occlusal, respectively) after gingival modelling with the retainer and the veneer tooth. The gingival line is aesthetically pleasing.

Six weeks after the decoronation surgery, a connective tissue autograft was used to increase mucosal thickness at the edentulous site (Figure 7). The tissue was harvested from the palate in the premolar region opposite to the edentulous site. Gingival bed preparation was initiated following a 3-week healing period. A vacuum-formed retainer was used to shape the soft tissue. To create a natural gingival profile [8,9,10], flowable composite (Cirus Flow, Elsodent, Cergy Pontoise, France) was successively added to the apical part of tooth # 13 located inside the retainer (Figure 7).

**Figure 8:**
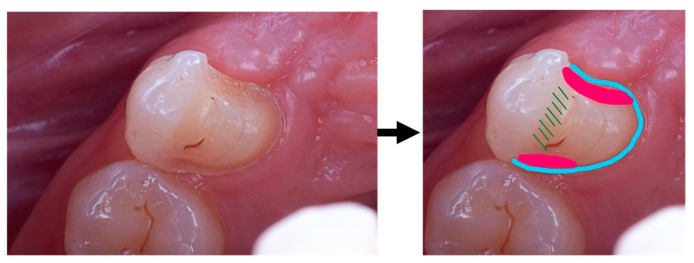
Cantilever bridge retainer design: blue—proximal and palatal chamfer; red—proximal boxes, with a larger box facing the edentulous region; green—homothetic reduction in the palatal cusp.

A premolar-supported cantilever bridge was chosen to replace tooth # 13 with a fixed, tooth-supported, minimally invasive prosthetic solution. Preparation of the abutment tooth required a 1.5 mm homothetic reduction in the palatal cusp of tooth # 14 [8,11], a partial peripheral chamfer (proximal and palatal), and mesial and distal boxes according to the preparation recommendations for bonded bridges [12] (Figure 8) (845KRD.314.025 and 8845KR.314.025 diamond burs, Komet, Dublin, Ireland).

**Figure 9:**
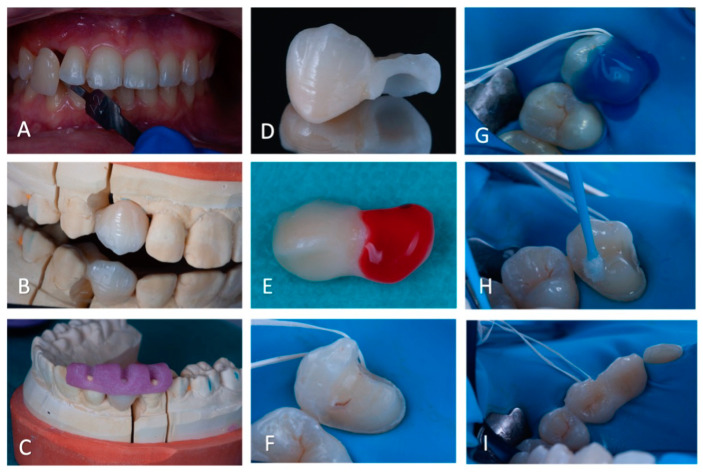
(**A**): Intra-oral photography displaying the visual colour assessment, using a shade-guide Table (**B**): Buccal view of the bridge positioned on the plaster model. (**C**): Physical plaster model with the resin repositioning key. (**D**): Buccal view of the cantilever bridge displaying the thickness of the retainer wing. (**E**): Etching of the intrados of the bridge. (**F**): Placement of the surgical field. (**G**): Etching of the supporting tooth. (**H**): Application of adhesive on the supporting tooth # 14. (**I**): Cantilever bridge immediately after bonding displaying the fit on the supporting tooth.

After colour recording using a shade guide (VITA Classical, VITA Zahnfabrik, Bad Sackingen, Germany) [13], a conventional impression was taken (Harmony, Elsodent, Cergy Pontoise, France). The prosthesis was then fabricated in the laboratory (Figure 9). Computer-aided design resulted in a 22 mm^2^ connection surface. A lithium disilicate-enriched glass-ceramic (LD, E.max Press MT, A2, Ivoclar-Vivadent, Schaan, Liechtenstein) framework was prepared. According to the manufacturer, LD can be used with a minimum connection area of 16 mm^2^.

A light-cured acrylic repositioning jig was fabricated to facilitate with the bonding of the bridge (Plaque Photo, Willmann & Pein Gmbh, Barmstedt, Germany) (Figure 9). After the fitting and validation of the shade and shape by the patient, the bonding sequence was performed.

A sectorial dam (Nictone thin, Bisico, France) was placed, avoiding to the greatest extent possible the rebound effect due to the tension of the dam in the edentulous zone [9] (Figure 9). The correct positioning of the bridge was checked again with the repositioning jig.

The intrados of the retainer wing was sandblasted (50 μm alumina sand, Airsonic Mini Sandblaster, Hager Werken, Duisburg, Germany) and then etched with 5% hydrofluoric acid (Ceramic Etch, VITA, Bad Sackingen, Germany) for 20 s. After rinsing and drying, silane (Monobond Plus, Ivoclar-Vivadent, Schaan, Liechtenstein) was applied for 1 min [8]. The repositioning jig was coated with glycerine (Liquid Strip, Ivoclar-Vivadent, Schaan, Liechtenstein).

After sandblasting, the supporting tooth was etched with orthophosphoric acid (G-Etch, Elsodent, Cergy Pontoise, France) for 15 s on the dentin and 30 s on the enamel. Due to the exposure of some dentinal areas on # 14, a three-step adhesive system (Syntac, Ivoclar-Vivadent, Schaan, Liechtenstein) was chosen (Figure 9). The two primers and the adhesive were applied according to the manufacturer’s recommendations [8].

The bonding was performed with a dual-cure composite resin (Variolink Esthetic, neutral, Ivoclar-Vivadent, Schaan, Liechtenstein) (Figure 8). The resin was light-cured for 20 s on each side (1600 mW/cm2, 385–515 nm, Valo, Ultradent) after placement of the prosthesis with its repositioning key. Polishing of the bonding resin was performed (Sof Lex, 3M, Saint Paul, MN, USA) and the occlusion was carefully checked (articulate paper, Bausch, Nashua, NH, USA).

**Figure 10:**
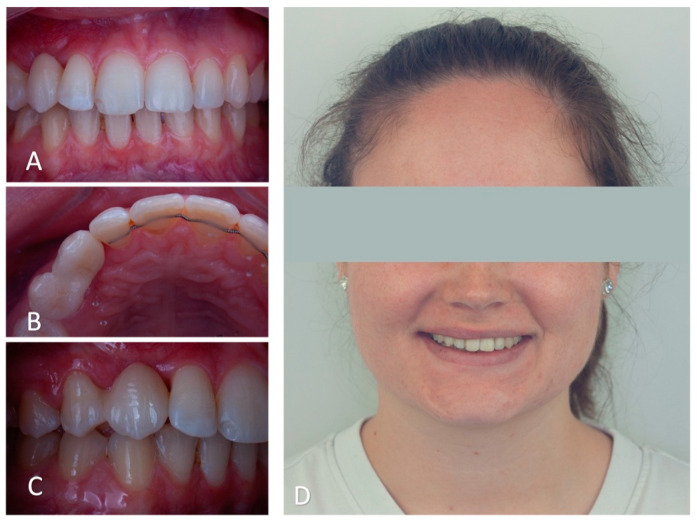
Immediate post-operatory images. (**A**): Intra-oral frontal view shows the black triangle between # 12 and # 13. (**B**): Occlusal view shows palatal cusp coverage of # 14 and the size of the connector. (**C**): Lateral intra-oral view of the bonded tooth # 13 displaying the gingival integration of the freshly bonded bridge. (**D**): Extra-oral frontal view of the patient’s smile, displaying the aesthetic integration of the cantilever bridge.

The patient was fitted with a night guard. At immediate post-operative assessment, a black triangle remained between # 12 and # 13 (Figure 10). The result was satisfactory for both the team and the patient (Figure 10).

**Figure 11:**
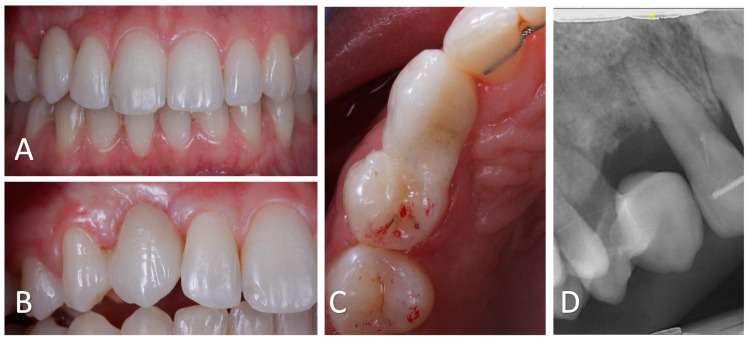
Follow-up at 19 months after surgery. (**A**): Intra-oral frontal view showing satisfactory aesthetics of the bonded bridge. The black triangle between # 13 and # 12 appears to be smaller. (**B**). Intra-oral lateral view displaying the healed gingiva in the cervical embrasure on both to the cantilever bridge. (**C**): Occlusal view of # 13 showing the absence of static or dynamic occlusal charge. (**D**): Sagittal CBCT radiograph showing bone preservation and partial replacement of the residual root by bone.

After 19 months of prosthetic rehabilitation, the patient is satisfied with the aesthetics of the prosthetic restauration (Figure 11). The cantilever bridge is well integrated both biologically and aesthetically. In addition, the prosthetic canine is free of occlusal charges (Figure 11). The periodontium is healthy and the bone appears to have been preserved (Figure 11). The black triangle between # 12 and # 13 appears to have decreased (Figure 11).

## 3. Discussion

The usual treatment options for the management of ankylosed maxillary canines are retention of the tooth in situ or tooth extraction [3]. In this case report, the patient presented with a severely infra-occluded maxillary right canine, with only 1.5 mm of the tip erupted. This situation presented a triple challenge in terms of aesthetics, functionality, and infection management.

An overlay was not feasible due to mechanical and biological constraints. Placement of a transcanine implant was also not possible as the tooth was not fully impacted, lacking soft tissue or bone coverage and lacking coronal bone height [14,15]. Extracting the tooth carried the risk of creating a significant bone defect and an unsightly gingival defect, because preservation of the buccal cortical bone was not guaranteed. Furthermore, even if the implant solution was not immediately considered, it was necessary to anticipate the potential need for future placement of a dental implant. Therefore, decoronation [6,16] was suggested in the current case.

Several therapeutic solutions are available [4,5,6]. In order to determine the optimal long-term prosthetic solution, the patient and clinician must carefully consider the advantages and disadvantages of removable, fixed, and implant-supported prostheses. Removable prostheses are usually not recommended due to their poor aesthetics, potential psychological impact, and increased risk of crushing the soft tissue of the edentulous area.

As a short-term solution, a removable retainer can be proposed [9]. In the present case, this temporary solution provided a rapid aesthetic improvement. In addition, the retainer’s intrados served as a healing guide and formed the gingival bed for the future prosthesis [8,10]. For short- and medium-term solutions, TOBBI (Temporary Orthodontic Bonding Bridge for Implant) bridges can also be suggested [19].

As for implant-supported rehabilitations, they may not be a viable option for medically compromised or younger patients [20]. The contraindication of implant treatment for young patients is supported by the fact that dental implants do not adapt to the residual vertical growth of the jaws or of the alveolar processes, nor the continuous eruption of adjacent natural teeth [20]. This could result in an infra-occlusion of the implant crown, as opposed to the crowns of the adjacent teeth, leading to aesthetic and functional issues. Similar observations of implant infra-position have been reported even in adult patients [21,22] with little or no active growth potential. As a result, placing an implant in the aesthetic zone for this patient could have caused aesthetic complications and could have potentially required the replacement of the implant-supported restoration. Therefore, it was decided to postpone implant placement.

As an alternative treatment, if the occlusal context is favourable and the periodontal support of the abutment teeth is sufficient, a conventional bridge can be proposed as a fixed prosthetic solution [23]. However, this option requires complete peripheral preparation of both supporting teeth, resulting in 50–70% tissue loss at the abutments and 10% risk of pulp vitality loss [9,24]. In this case, the apical root resorption of tooth # 12 and the low interdental alveolar septum distal to tooth # 12 contraindicated this treatment [23].

Last but not least, bonded bridges have been proposed for years as a minimally invasive solution with maximum tissue preservation [9,12,25,26]. Their evolution from two-wing to single-wing bridges and from metal–ceramic to ceramic restorations has been a technical revolution. In recent years, the single retainer bridge (also known as a cantilever bridge) has been developed with either a zirconia (ZR) or a lithium disilicate vitreous ceramic (LD) frame [8,10,12,27]. Currently, ceramic-bonded cantilever bridges improve patients’ quality of life, especially in the absence of medium- and long-term post-operative complications [28]. However, the preparation of the supporting tooth plays an important role in the mechanical strength of cantilever bridges [24,27]. On incisors and canines, the supporting tooth preparations include a cervical ridge, an occlusal ridge, a proximal connection box opposite the edentulous zone, and a macrowell [8,10,12,27]. In the posterior region, several retainer designs have been proposed [24], and Kasem et al. [24] described a complete coverage of the lingual cusps on the supporting teeth. This design allows an optimal distribution of occlusal forces, with their concentration in the proximal box and above the cemento-enamel junction of the supporting tooth [24]. Thus, in the present clinical case, the palatal cusp of tooth 14 was covered (Figure 8). This preparation increases the bonding surface and allows mechanical retention and stability of the prosthesis [9].

The literature presents limited data on the survival of bonded cantilever bridges used to replace a posterior tooth or a canine [9,12,26]. Still, in a randomised controlled clinical trial [25], a 97.1% success rate after an average follow-up of 23 months for metal–ceramic cantilever bridges with a wrap-around design replacing a molar was reported. Another study reported a success rate of 96.3% after 53 months of follow-up for cuspid-covering ZR cantilever bridges replacing posterior teeth from canines to molars [9]. Therefore, the survival prognosis of the cantilever bridge in the present case is favourable.

LD was chosen due to its excellent aesthetic and mechanical properties, as well as its good bonding potential [27,29,30]. Indeed, the fracture resistance of ZR is superior to that of LD. Yet, in vitro tests show the better adhesion of LD to a dental substrate [13,29]. As a consequence, for anterior cantilever bridges, glass-ceramic restorations have a higher success rate than ZR [29]. However, for anterior LD bridges, the connector surface should be increased and should be at least 12 mm^2^ or even 16 mm^2^ [8]. By contrast, if a ZR frame is chosen, the connection surface must be at least 9 mm^2^ [12]. In this case report, the connection surface was 22 mm^2^, which was considered favourable for the LD framework, providing sufficient surface area for stability and load distribution. A 1.5 mm homothetic reduction in the lingual cusp was performed according to the preparation recommendations for bonded partial restorations [8,11]. This provided an optimal biomaterial thickness of the retainer on the supporting tooth (Figure 9).

While presenting a minimally invasive solution for single missing teeth, cantilever bridges are subjected to biomechanical complications [31]. Debonding might occur if the bonding procedures are not respected [31]. In this case, the bonding procedure has been thorough and according to manufacturer recommendations. Furthermore, debonding is more frequent for zirconia bridges compared to disilicate bridges [31]. In disilicate cantilever bridges, catastrophic fracture might occur, especially in cases of high occlusal forces [31]. However, in this clinical case, the occlusion was optimised to avoid static and dynamic load on the cantilever canine, thus avoiding fracture [31]. To further enhance the survival of the cantilever bridge, the patient should avoid biting on excessively hard foods and should regularly visit the dentist for an examination. Indeed, annual examinations allow the early diagnosis of fracture or debonding [9,23,24]. In addition, regular control and maintenance of good oral hygiene is important to avoid biological risks like caries and periodontitis [31,32]. In fact, 5 years after rehabilitation with a cantilever bridge, carious lesions are found in 1.5% of cases and periodontal disease in 2.1% of cases [32].

Another concern with the prosthodontic rehabilitations is the wear of the prosthesis and the wear of the antagonist teeth. While a rough lithium disilicate surface can excessively wear a natural antagonist, tooth wear caused by a lithium disilicate prosthesis is comparable to the tooth wear of natural teeth [33].

Another risk that requires regular monitoring is the risk of orthodontic relapse. If an upper bonded retainer maintains the position of the upper incisors, a diastema between teeth # 12 and # 13 may develop in the long term [23]. A contact point displacement between # 13 and # 12 could also occur if the supporting tooth, # 14, presents a rotation relapse. However, in the present case, the occlusal context is reassuring [23]. Furthermore, if the patient is compliant in the wearing of the bite-guard, the risk of orthodontic relapse is reduced [9].

Like any treatment plan, this one has its limitations. The presence of a small residual black triangle between teeth # 12 and # 13 is one of them. The reduced size of the papilla was anticipated in the beginning and was due to a reduced bone level. This drawback could have been limited with additional orthodontic treatment. However, invasive and risky treatment would have been necessary to improve this minor aesthetic inconvenience. For instance, in order to apically move the # 12 to # 13 contact point, thus improving the interdental papilla, stripping of the distal side of the # 12 tooth would have been necessary. This would have inevitably reduced the mesio-distal diameter of # 12 compared to # 22, leading to unpleasant smile aesthetics. Alternatively, further distal tilt of the # 12 root could have improved the situation of the inter-dental papilla as well. This treatment option has been excluded, as the # 13 and # 12 root were already fairly close to each other (Figure 2). For instance, further apical movement of the lateral incisor would have mandated its coronoplasty, as the incisal edge would have been placed lower than the aesthetic plane. Thus, the multidisciplinary team has decided against further orthodontic treatment because of the pre-existing root resorptions. Indeed, continuing orthodontic treatment could have worsened the existing root resorptions, therefore exposing the patient to the risk of premature tooth loss [7]. For the same reasons, orthodontic treatment has not considerably enhanced the bone architecture. In fact, orthodontic extrusion can facilitate bone remodelling and enhance the overall success of any prosthodontic rehabilitation. However, in this case, any further bone, gum, or occlusion optimisation could have been detrimental to the patient’s health and would have implied invasive treatment [7]. After an initial failure of the orthodontic treatment in another institution because of the ankylosed canine, the orthodontic treatment time was deemed exceedingly long and presented long-term risks. As a consequence, the treatment objectives were limited to the correction of the occlusal plane cant and prosthodontic rehabilitation of # 13, leaving the sagittal malocclusion untreated.

Regardless of the difficulty of this treatment plan, the final outcome has long-term functionality and warrants good aesthetics for the patient.

Although bonded bridges to replace canines have not been extensively described in the literature, it is imperative that the existing preparation recommendations and the minimum thicknesses of the biomaterials are followed. Further studies are required before this management can be systematised for canine replacement.

## 4. Clinical Significance

This clinical case highlights the importance of multidisciplinary management in dentistry. In the case of an ankylosed canine in a young patient, with multiple root resorptions and an occlusal cant, a compromise treatment had to be chosen. The development of the prosthetic space and the harmonisation of the smile line, the preservation of the bone capital, and the preparation of the gingival bed allowed an aesthetic and functional rehabilitation, even in the compromise treatment.

The treatment with a cantilevered bonded bridge has enabled an aesthetic and minimally invasive rehabilitation. This technique is more cost efficient than implants, is reversible, does not affect pulp vitality, and is a viable solution for rehabilitating the smiles of young patients.

## Data Availability

The data that support the findings of this study are available from the corresponding author, T.R., upon reasonable request.
